# A national health facility survey of malaria infection among febrile patients in Kenya, 2014

**DOI:** 10.1186/s12936-016-1638-2

**Published:** 2016-12-08

**Authors:** Sophie Githinji, Abdisalan M. Noor, Josephine Malinga, Peter M. Macharia, Rebecca Kiptui, Ahmeddin Omar, Kiambo Njagi, Ejersa Waqo, Robert W. Snow

**Affiliations:** 1 KEMRI-Wellcome Trust Collaborative Programme, Nairobi, Kenya; 2 Centre for Tropical Medicine and Global Health, Nuffield Department of Clinical Medicine, University of Oxford, Oxford, UK; 3 National Malaria Control Programme, Ministry of Health, Nairobi, Kenya

## Abstract

**Background:**

The use of malaria infection prevalence among febrile patients at clinics has a potential to be a valuable epidemiological surveillance tool. However, routine data are incomplete and not all fevers are tested. This study was designed to screen all fevers for malaria infection in Kenya to explore the epidemiology of fever test positivity rates.

**Methods:**

Random sampling was used within five malaria epidemiological zones of Kenya (i.e., high lake endemic, moderate coast endemic, highland epidemic, seasonal low transmission and low risk zones). The selected sample was representative of the number of hospitals, health centres and dispensaries within each zone. Fifty patients with fever presenting to each sampled health facility during the short rainy season were screened for malaria infection using a rapid diagnostic test (RDT). Details of age, pregnancy status and basic demographics were recorded for each patient screened.

**Results:**

10,557 febrile patients presenting to out-patient clinics at 234 health facilities were screened for malaria infection. 1633 (15.5%) of the patients surveyed were RDT positive for malaria at 124 (53.0%) facilities. Infection prevalence among non-pregnant patients varied between malaria risk zones, ranging from 0.6% in the low risk zone to 41.6% in the high lake endemic zone. Test positivity rates (TPR) by age group reflected the differences in the intensity of transmission between epidemiological zones. In the lake endemic zone, 6% of all infections were among children aged less than 1 year, compared to 3% in the coast endemic, 1% in the highland epidemic zone, less than 1% in the seasonal low transmission zone and 0% in the low risk zone. Test positivity rate was 31% among febrile pregnant women in the high lake endemic zone compared to 9% in the coast endemic and highland epidemic zones, 3.2% in the seasonal low transmission zone and zero in the low risk zone.

**Conclusion:**

Malaria infection rates among febrile patients, with supporting data on age and pregnancy status presenting to clinics in Kenya can provide invaluable epidemiological data on spatial heterogeneity of malaria and serve as replacements to more expensive community-based infection rates to plan and monitor malaria control.

## Background

The World Health Organization (WHO) recommends that all persons suspected of malaria should be examined for evidence of Plasmodium infection by either microscopy or rapid diagnostic tests (RDTs) before treatment is initiated [1]. RDTs for *Plasmodium falciparum* malaria are known to provide accurate diagnosis within a few minutes [2–5] and antimalarial treatment can be safely withheld if the result is negative [6]. Since the adoption of this global policy in 2010, the use of RDTs has doubled in Africa and increased the proportion of suspected malaria cases receiving a diagnostic test from 47 to 62% in 2013 [7]. Kenya adopted the policy on universal parasitological diagnosis on all cases suspected of malaria in 2010 [8] and in 2012, the National Malaria Control Programme (NMCP) embarked on a plan of rolling out RDTs to strengthen the capacity of malaria diagnostic services across the country [9].

The use of fever test positivity rates (TPR) has a long history as a measure of malaria risk in communities, most notably those in areas where the ambition is malaria elimination [10, 11]. More recently, health facility-based surveys of malaria infection prevalence in febrile patients have been used as part of the rapid analysis of malaria risks in urban settings in Angola, [12] Mozambique [13], Burkina Faso, Benin, Tanzania and Côte d’Ivoire [14]. They have also been used as part of national surveys of malaria epidemiology in Niger [15] and The Gambia [16], and as a means to operationally measure intervention effectiveness through sentinel based case-control studies in Benin [17] and Madagascar [18].

Quality of malaria diagnosis and treatment studies have been undertaken in Kenya on a bi-annual basis since 2010 [19–21]. These surveys have focused on describing routine clinical practices among febrile patients presenting to government out-patient departments and have highlighted that while the practice of parasitological diagnosis has increased significantly since 2010, testing rates for malaria have remained suboptimal. Over 30% of patients with fever were not tested with either an RDT or a blood slide in health facilities where diagnostics were available in September 2014, the month preceding the survey. This could be attributed to health worker clinical practices. Malaria case management trainings emphasizing on testing before treatment and routine supervisory visits have been recommended to further improve adherence to the policy [20]. The present study aimed to characterize the TPR of malaria in patients with reported fever presenting to clinics across Kenya to define the patterns of febrile infection rates nationwide as a potential epidemiological surveillance tool, not possible from routine data or from standard quality of care surveys.

## Methods

### Study design and sampling

A national cross-sectional survey was undertaken at public health facilities sampled according to malaria endemicity zones. A stratified sampling frame was developed based upon the universe of 4242 geo-coded public health facilities offering out-patient general clinical services [22, 23] within five malaria epidemiological zones. These zones have been used by the Ministry of Health for over 40 years [24, 25] and refined more recently to provide targeted malaria control services [22, 26, 27]. These malaria zones cover the high, stable perennial transmission areas of western Kenya around Lake Victoria (lake endemic), moderate, seasonal transmission areas along the Kenyan coast (Coast endemic), acutely seasonal, low transmission areas of northern, eastern and southeastern Kenya (seasonal low transmission), the unstable, variable transmission areas of the highlands west of the Rift Valley (highland epidemic) and the central highlands including areas around Nairobi traditionally considered either free of malaria or of exceptionally low transmission (low risk).

**Fig. 1:**
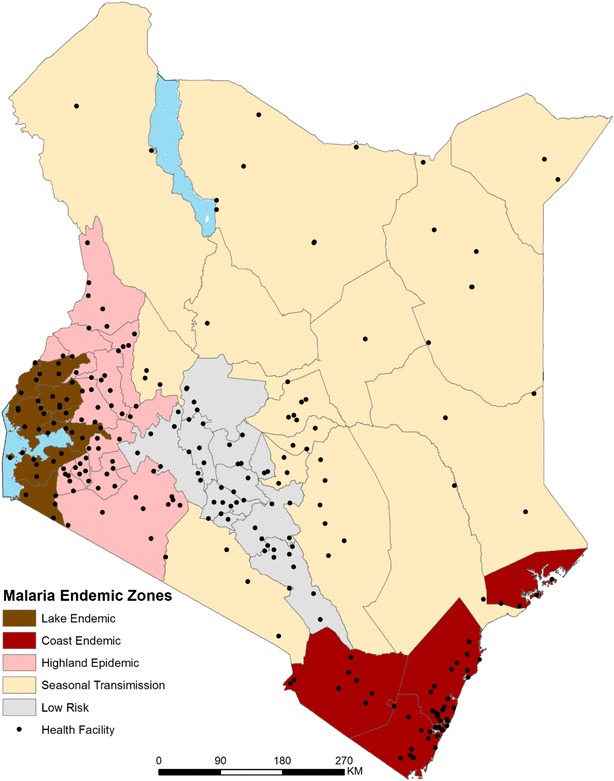
Location of sampled health facilities across five malaria endemicity zones in Kenya

The sample size was determined based on the proportion of positive *P. falciparum* obtained during the RDT piloting period from 2007 to 2010, aggregated by endemicity zone. This proportion was used to compute the number of fever cases necessary to provide the estimated malaria case load per zone. A spatial sampling design tool was then used to draw a random spatially weighted sample proportionate to number and types of health facilities within each zone (Fig. 1). With a target of at least 50 febrile patients per facility [28], 234 public health facilities were required across all zones.

### Data collection

The survey was conducted in October and November 2014, coinciding with the short rainy season. Seventy-six senior laboratory technologists underwent a two day training workshop on survey procedures and a refresher course on how to diagnose malaria using *Carestart* HRP2 *P. falciparum* RDT. Each survey technologist was based at a sampled facility for a maximum of 5 days to recruit all out-patients reporting any history of fever in the last 48 h identified by clinicians who screened all presenting patients on the survey days. Patients with a history of fever in the last 48 h, who had not sought anti-malarial treatment before attending the facility were asked to provide informed consent. For each consenting participant, information was collected on basic demographics and usual residence. For women aged between 15 and 49 years, additional information on pregnancy status was recorded. Each patient provided a single finger-prick blood sample for the malaria RDT. The RDT results were read as per the manufacturer’s instructions and recorded separately on the patient’s questionnaire. Test results were then passed on to the health care provider to use in managing the patient.

### Data management and analysis

Data entry was undertaken in Access (Microsoft, USA) using customized data entry screens with inbuilt consistency checks. Data analysis was performed using STATA, version 12 (StataCorp, College Station, Texas, USA). The primary outcome variable was prevalence of malaria calculated as a percentage of the total number of people tested that had RDT positive results. Analysis was undertaken overall and per malaria risk zone.

## Results

### Sample description

The survey was conducted at all 234 sampled health facilities, comprising of 23 hospitals, 99 health centres and 112 dispensaries (Table 1). A total of 58,080 out-patients presented at the health facilities during the survey days and 12,913 (22.2%) reported a history of fever within the last 48 h. 10,557 consenting febrile out-patients without previous anti-malarial treatment were enrolled into the survey. Thirteen patients, all of them with negative RDT test results, were excluded from the analysis because of missing data on age. The background characteristics of the surveyed patients by malaria risk zone are shown in Table 1.

**Table 1:** Background characteristics of 10,544 surveyed patients from 234 public health facilities in Kenya 2014

Background characteristic	Lake endemic N = 1847	Coast endemic N = 2163	Highland epidemic N = 2213	Seasonal low transmission N = 2247	Low risk N = 2074	Total N = 10,544
Patients						
Age (years)						
<5	606	794	634	471	534	3039
5–14	525	581	524	461	410	2501
≥15	716	788	1055	1315	1130	5004
Male	774	956	928	929	787	4374
Female	1073	1207	1285	1318	1287	6170
Pregnant	51	77	70	126	108	432
Facility type						
Total facilities	37	47	50	54	46	234
Hospital	5	5	3	5	5	23
Health centre	12	11	22	27	27	99
Dispensary	20	31	25	22	14	112

### Prevalence of malaria by malaria zone and age

Overall, 1633 (15.5%) of the febrile patients surveyed were RDT positive for malaria at 124 (53.0%) facilities. Among non-pregnant patients 1600 (15.8%) infections were detected among 10,112 patients with a recent history of fever. As would be expected, infection prevalence among non-pregnant patients varied between malaria risk zones, ranging from 0.6% in the Low risk zone to 41.6% in the high Lake endemic zone (Table 2). At 110 (47%) facilities, no patients were found to be RDT positive during the survey, of these 38 (34.5%) were located in the low risk zone, and the proportion of facilities without any RDT positive febrile patients followed a similar pattern to the fraction of positive patients in each malaria zone (Table 2).

**Table 2:** RDT prevalence of infection among patients with reported fever in last 48 h at public health facilities in Kenya 2014 – n/N (%) [95% confidence interval]

	Lake endemic	Coast endemic	Highland epidemic	Seasonal low transmission	Low risk	Total
Mean^a^	41.30%	21.90%	13.10%	3.80%	0.60%	14.70%
Median	46.00%	10.00%	2.00%	0.00%	0.00%	2.00%
IQR	31.0–56.0%	2.1–46.0%	0.0–19.0%	0.0–2.3%	0.0–0.0%	0.0–22.1%
Proportion of facilities without any RDT positive fevers	0/37 (0%)	10/47 (21.3%)	22/50 (44.0%)	40/54 (74.1%)	38/46 (82.6%)	110/234 (47.0%)
Percentage positive by age group						
<1 year	42/131 (32.1%) [24.2–41.1]	6/176 (3.4%) [1.3–9.1]	9/122 (7.4%) [3.5–14.8]	2/108 (1.9%) [0.4–7.6]	0/147 (0%)	59/684 (8.6%) [6.2–11.9]
1–4 years	253/476 (53.2%) [44.0–62.1]	133/618 (21.5%) [13.9–31.6]	75/512 (14.6%) [8.5–24.1]	15/363 (4.1%) [1.3–12.5]	4/387 (1.0%) [0.4–2.6]	480/2356 (20.4%) [16.5–25.0]
5–9 years	186/286 (65.0%) [57.5–71.9]	146/357 (40.9%) [31.0–51.6]	66/284 (23.2%) [13.5–37.1]	13/264 (4.9%) [1.6–14.5]	0/247 (0%)	411/1438 (28.6%) [23.5–34.3]
10–14 years	133/239 (55.6%) [46.6–64.4]	92/224 (41.1%) [29.3–54.0]	60/240 (25.0%) [14.2–40.1]	12/197 (6.1%) [2.2–15.7]	0/163 (0%)	297/1063 (27.9%) [22.7–33.9]
15+ (non-pregnant)	134/664 (20.2%) [15.7–25.6]	93/711 (13.1%) [8.9–18.7]	88/985 (8.9%) [5.3–14.8]	31/1189 (2.6%) [1.3–5.1]	7/1022 (0.7%) [0.3–1.4]	353/4571 (7.7%) [6.2–9.6]
TPR for all non-pregnant patients	748/1796 (41.6%) [36.1–47.4]	470/2086 (22.5%) [16.0–30.8]	298/2143 (13.9%) [8.5–21.9]	73/2121 (3.4%) [1.6–7.4]	11/1966 (0.6%) [0.3–1.1]	1600/10,112 (15.8%) [13.0–19.1]
Pregnant women	16/51 (31.4%) [17.6–49.4]	7/77 (9.1%) [3.8–20.0]	6/70 (8.6%) [3.6–18.9]	4/126 (3.2%) [1.2–7.4]	0/108 (0%)	33/432 (7.6%) [5.1–11.4]
Percentage of positives aged						
<1	42/764 (5.5%) [3.9–7.6]	6/477 (1.3%) [0.5–3.3]	9/304 (3.0%) [1.4–6.0]	2/77 (2.6%) [0.9–7.5]	0 (0%)	59/1633 (3.6%) [2.7–4.8]
<5	295/764 (38.6%) [33.0–44.6]	139/477 (29.1%) [23.2–36.0]	84/304 (27.6%) [23.2–32.5]	17/77 (22.1%) [10.8–39.8]	4/11 (36.4%) [19.5–57.5]	539/1633 (33.0%) [29.6–36.6]
RDT Test Positivity Rate	764/1847 (41.4%) [35.8–47.1]	477/2163 (22.1%) [15.6–30.2]	304/2213 (13.7%) [8.4–21.6]	77/2247 (3.4%) [1.6–7.4]	11/2074 (0.5%) [0.3–1.1]	1633/10,544 (15.5%) [12.7–18.6]

^a^Refers to the mean between health facilities in each zone

**Fig. 2:**
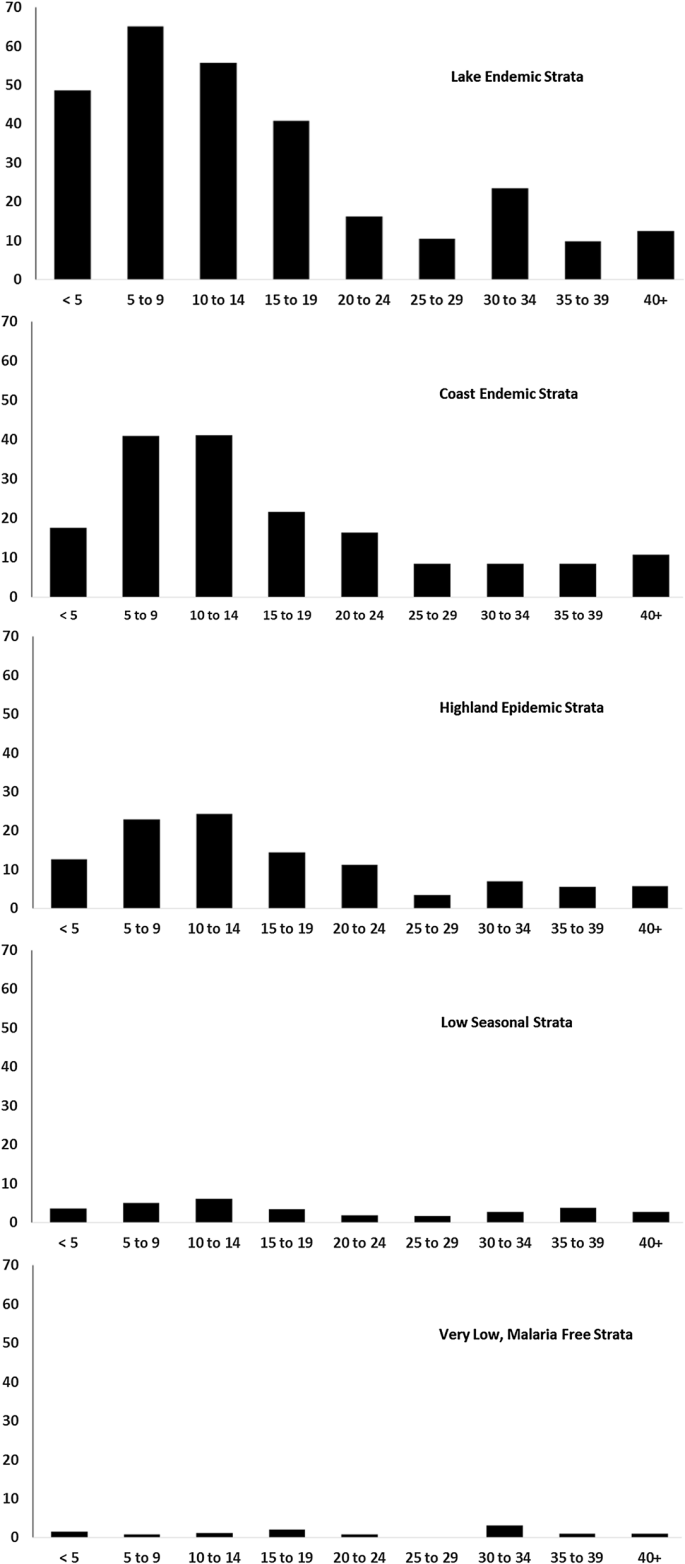
Age-specific test positivity rates among febrile patients attending out-patient clinics in October–November 2014 in five malaria endemicity zones

The age pattern of infection among non-pregnant patients across all malaria zones followed a similar pattern, with low infection rates in febrile infants rising to the highest infection rates among febrile children aged 5–9 and/or 10–14 years, then declining through young adulthood (Fig. 2). There were, however, important differences in age positivity between malaria zones. The typical age-pattern of infection was much less pronounced in the very low risk zone, with risks of infection among fevers being similar across most age groups outside of infancy. Infants in the lake endemic zone had the highest rates of infection compared to all the other endemicity zones (Fig. 2). In the lake endemic zone, 6% of all infections were among children aged less than 1 year, compared to 3% in the coast endemic and highland epidemic zone and less than 1% in the low risk zone and 0% in the exceptionally low risk zone (Table 2).

### Malaria infection among febrile pregnant women

A total of 432 women with a recent history of fever reported being pregnant at the time of the consultation, of whom 33 (7.6%) were RDT positive. Sixteen of these infections were identified in the lake endemic zone among 51 pregnant women (prevalence 31.4%). Infection prevalence among febrile pregnant women was less than 10% in the coast endemic and highland epidemic zone, 3.2% in the seasonal low transmission zone and zero in the exceptionally low risk zone (Table 2).

## Discussion

The present study was not designed to compare the direct congruence between fever test positivity rates (TPR) at health facilities and the prevalence of malaria infection among communities served by these facilities. Rather, a comparison is made between RDT positivity among all fevers presenting to facilities against the operational definitions of malaria zones used by the NMCP to define intervention options for control. The overall TPR (Table 2), and the age-patterns of TPR recorded at public health facilities (Fig. 2), varied between malaria endemic zones in ways which could be used to operationally define malaria risk in communities and importantly how these age-specific metrics change between seasons, between years and overtime as interventions to prevent malaria exposure increase in coverage. The traditional high transmission areas around Lake Victoria showed the highest overall TPR, the highest rates of infection among febrile infants and the highest rates among febrile pregnant women (Table 2; Fig. 2). Areas that the Kenyan Ministry of Health regards as having intermediate transmission along the Kenyan coast or in the epidemic prone areas of the highlands correspond to lower levels of overall TPR with fever infection rates less concentrated in the youngest children and intermediate levels of infection among febrile pregnant women (Table 2; Fig. 2). Areas where transmission has been historically very low are characterized, as one might expect, with very low levels of infection among fevers presenting to clinic in all age groups or no infection reported in infants or pregnant women (Fig. 2).

These patterns of infection in fevers across the varied epidemiology of Kenya are important because the default for classifications of malaria risk zones in Africa continues to be community-based malaria infection cross-sectional surveys [29]. Modelled and mapped community-based parasite prevalence is used for planning control across Africa [29–31] and to subsequently model presumed malaria burdens [7]. Surveys among asymptomatic individuals at the community level are expensive, even when constrained to simpler sampling frames such as school children [32]. Conversely, the testing of all fevers at health facilities should be routine [8] and in theory these data should be available at no additional cost. Routine continuous data from facilities have the additional advantage of covering every month of every year, as opposed to single snap shot data from one off malaria indicator surveys. If data were available with enough collateral information on age, pregnancy status and facilities were geo-coded, such data would provide invaluable epidemiological evidence for programmes to compute spatial epidemiological risks to plan and monitor control operations and ultimately migrate into a surveillance system able to identify “hot spots” for targeted disease control [33] and be useful to exclude malaria risks (for example by examining infant or pregnancy fever infection risks, Table 2).

In The Gambia a more direct comparison of malaria infection rates among fevers at clinics and corresponding infection and serological rates at matched communities showed very similar age-patterns of risk and spatial heterogeneity [16]. A study of infection prevalence among fevers at clinics, similar to the study presented here, undertaken across Niger between 2009 and 2010, showed a congruence with the established bio-climatic zones and seasonality used by the Niger National Malaria Control Programme to target malaria control [15]. Importantly, the Niger study highlighted the inadequacy of routine data from the health information system which included those suspected and not confirmed with malaria and the incomplete nature of data on how many individuals were tested for malaria [15].

There have been significant investments across Africa, including Kenya, to improve the ability of routine services to accurately record health information in a timely fashion through the District Health Information System 2.0 (DHIS2.0) or the Integrated Disease Surveillance Reporting System (IDSR) [34–36]. However, the detail, completeness and coverage of these data necessary to provide reliable epidemiological data for malaria programmes remains poor [37]. In Kenya, not all fevers are tested [20], the numbers of people tested is not always recorded, data are aggregated over districts, age groups, and time losing the granularity of information on age, seasonality, location and pregnancy status [38].

Interestingly, there was a high frequency of infection among children aged 10–14 years of age presenting with fever to clinics in all endemicity zones (Fig. 2). Recent studies across different settings have reported a gradual shifting of peak parasite prevalence of malaria from younger to older children [39–41]. This has been attributed to enhanced control efforts that have resulted in children acquiring immunity to malaria more gradually than in the past and clinical attacks occurring in school-age children more frequently [42]. This school-aged population are neglected from most child clinic services and community-based vector control programmes [42–44], however they are an ever important source of clinical malaria infections that should be highlighted during future clinical training programmes.

The default diagnostic test used in the present study was RDTs, these are far more ubiquitous than microscopy in Kenya [21] are available at all levels of the health sector and are subject to less between observer variability [2]. Patients who reported taking any anti-malarial treatment in the 2 weeks preceding the survey were excluded from the study because of the well-known persistence of HRP2 antigenaemia after treatment [45]. However, a recent study in Uganda reported a much longer HRP2 persistence period extending to a median of 35 to ≥42 days after treatment [46]. It is, therefore, possible that patients found positive in this survey, especially in the high endemic areas, may have been due to persistent HRP2 antigenaemia. However, the associated risk of overtreatment of uninfected cases is considered more acceptable in malaria endemic zones than taking the risk of failing to detect cases. Recently, there has been reports of deletion of HRP2 gene which may lead to false RDT negative results [47, 48]. However, studies conducted in Africa showed HRP2 deletion in very small numbers of parasite isolates [49, 50], hence highly unlikely that the phenomenon may have influenced the results of this survey. The aim of the survey reported here was not to measure “true” prevalence of malaria in febrile populations, through expert microscopy or polymerase chain reaction (PCR) assays, but emulate what might be routinely available if collected on all febrile patients. This study was limited in that it was restricted to only one time of the year and, therefore, not representative of periods of low transmission. Nor did the study examine the effects of residence and travel time to facilities that may independently of age affect TPR [51]. A more detailed analysis of pockets of high transmission within broad ecological zones would have required larger facility sample sizes, beyond the scope of the present study. Pregnancy status was only asked about and no tests were done. This could have caused certain underreporting of malaria infection in pregnancy. Finally, it would have been interesting to examine a more direct spatial and temporal matched congruence between facility-based TPR and community-based parasite prevalence across the country. Such studies would enable a calibration between two dominant measures used in malaria risk mapping and a more reliable pathway to estimating the relationships between combinations of TPR and community based prevalence with disease incidence [52–54].

## Conclusions

Malaria infection rates among febrile patients presenting to clinics in Kenya could provide invaluable epidemiological data on the seasonal, temporal and spatial heterogeneity of malaria and serve as replacements to more expensive community-based infection rates to plan and monitor malaria control. However, for these data to be of use for the national malaria control programme, increased testing rates, more granular information on patient level data and improved coverage of routine data are required.
